# Sonic hedgehog (SHH) signaling improves the angiogenic potential of Wharton’s jelly-derived mesenchymal stem cells (WJ-MSC) 

**DOI:** 10.1186/s13287-017-0653-8

**Published:** 2017-09-29

**Authors:** Gabriela Zavala, Catalina P. Prieto, Andrea A. Villanueva, Verónica Palma

**Affiliations:** Laboratory of Stem Cells and Development, Faculty of Sciences, Universidad de Chile, Las Palmeras 3425, Ñuñoa, 7800003 Santiago, Chile

**Keywords:** Angiogenesis, WJ-MSC, Sonic hedgehog, Umbilical cord, Niche factor, VEGF, CAM assay

## Abstract

**Background:**

Wharton’s jelly-derived mesenchymal stem cells (WJ-MSC) show remarkable therapeutic potential to repair tissue upon injury via paracrine signaling by secreting diverse trophic factors that promote angiogenesis. However, the mechanisms and signaling pathways that regulate the induction of these specific factors are still mostly unknown. Emerging evidence suggests that Sonic hedgehog (SHH) plays a central role in angiogenesis and tissue maintenance. However, its contribution to the angiogenic potential of MSC has not been fully addressed. The aim of this work was to characterize the expression of the SHH pathway components in WJ-MSC primary cultures and to evaluate their angiogenic responsiveness to SHH signaling.

**Methods:**

Primary cell cultures obtained from human umbilical cords were treated with pharmacological modulators of the SHH pathway. We evaluated the modulation of diverse trophic factors in cell lysates, conditioned medium, and functional in vitro assays. In addition, we determined the angiogenic potential of the SHH pathway in the chicken chorioallantoic membrane, an in vivo model.

**Results:**

Our results show that WJ-MSC express components of the canonical SHH pathway and are activated by its signaling. In fact, we provide evidence of basal autocrine/paracrine SHH signaling in WJ-MSC. SHH pathway stimulation promotes the secretion of angiogenic factors such as activin A, angiogenin, angiopoietin 1, granulocyte-macrophage colony-stimulating factor, matrix metallometallopeptidase -9, and urokinase-type plasminogen activator, enhancing the pro-angiogenic capabilities of WJ-MSC both in vitro and in vivo.

**Conclusion:**

WJ-MSC are a cell population responsive to SHH pathway stimulation. Basal SHH signaling is in part responsible for the angiogenic inductive properties of WJ-MSC. Overall, exogenous activation of the SHH pathway enhances the angiogenic properties of WJ-MSC, making this cell population an ideal target for treating tissue injury.

**Electronic supplementary material:**

The online version of this article (doi:10.1186/s13287-017-0653-8) contains supplementary material, which is available to authorized users.

## Background

Mesenchymal stem cells (MSC), first described in the bone marrow, are multipotent and have the potential to differentiate into mesodermal lineages [1, 2]. They display low immunogenicity and have anti-inflammatory [3] and trophic properties [4], indicating that paracrine factors may play a key role in MSC-mediated modulation of acute and chronic pathological conditions [5, 6]. MSC are located in the perivascular niche surrounding blood vessels and form part of diverse tissues in the body, such as the bone marrow, adipose tissue, and embryonic annexes, among others [7–9]. The umbilical cord (UC) is an ideal model because its blood vessels (two arteries and one vein) are delimited by the Wharton’s jelly (WJ), a gelatinous substance that offers structural support and is densely populated by MSC [10], called Wharton’s jelly-derived MSC (WJ-MSC) [11, 12]. WJ-MSC secrete a rich panel of trophic factors, or secretome [13–15], which is especially enriched with angiogenic factors and promotes angiogenesis both in vitro, shown via tubule formation assays, and in vivo, demonstrated with the chicken chorioallantoic membrane (CAM) assay [13–15]. Accordingly, the pro-angiogenic competence of WJ-MSC makes them attractive for use in regenerative medicine due to their potential application for chronic injury wound healing [15–17] and cardiac repair [18–20], among other domains. Thus, it is necessary to understand how the angiogenic inductive properties of WJ-MSC are regulated and how different signaling pathways interact to enhance this angiogenic response.

Sonic hedgehog (SHH) is a morphogen that plays a fundamental role during mammalian embryonic development, mediating essential tissue patterning events. In postembryonic stages, SHH is important for the maintenance of homeostatic processes such as angiogenesis and cardiac repair, among others [21, 22]. SHH is secreted as a monomer, multimer, or in exovesicles [23], and exerts its cellular function through a molecular machinery located in the primary cilium [24–26]. Once SHH binds to its 12-pass transmembrane receptor Patched 1 (PTCH1) [27], it unleashes Smoothened (SMO) from its inhibition. SMO is a 7-pass transmembrane receptor, a member of the G protein-coupled receptor (GPCR) superfamily which, once released, transduces the SHH signal through the cytoplasm [28, 29] resulting in the activation of the zinc-finger transcription factors of the GLI family: GLI1, GLI2 and, to a lesser extent, GLI3. GLI factors recognize GLI binding sites (GBS) in DNA to stimulate the transcription of target genes, including *PTCH1* and *GLI1*, via negative and positive feedback loops, respectively [30, 31].

Although early researchers considered SHH to be active in embryos and absent, or silent, in adults [21, 32], recent data have recognized SHH as an active pathway during adulthood in pathological processes such as cancer [33–36] or during tissue repair [37–39]. Notably, SHH signaling not only plays a role in embryonic angiogenesis [40, 41] but also in adult tissue neovascularization. In fact, SHH signaling exerts indirect angiogenic modulation of the stroma that surrounds the blood vessels. In this microenvironment, SHH influences stromal cells in the perivascular niche to induce the expression and secretion of pro-angiogenic factors, which in turn act in the vasculature to induce the formation of new vessels to enhance the healing processes [38]. It has recently been shown that noncanonical SHH signaling, which is independent of transcriptional changes mediated by GLI transcription factors, directly promotes blood vessel formation. More specifically, it acts on the cytoskeleton of endothelial cells by modulating PI3-kinase [42] and the small monomeric GTPases RhoA and Rac1 [43–49]. These findings highlight the central role of the SHH pathway in diverse angiogenic processes.

Within the context of vascular repair, WJ-MSC have been shown to be effective in regenerative medicine therapies [18, 50]. Nevertheless, most of the biology and mechanisms by which perivascular WJ-MSC function are still unknown. Since the SHH ligand is one of the most highly expressed genes in the umbilical cord, along with other components of the pathway [46, 47], we sought to determine: 1) whether the angiogenic potential of WJ-MSC is modulated by the canonical SHH signaling pathway; and 2) whether the pro-angiogenic properties of WJ-MSC are mediated by the basal autocrine signaling of the SHH pathway in these cells. We show that SHH is expressed in WJ-MSC and exerts an autocrine signal on WJ-MSC to induce angiogenic factor secretion. Thus, we propose that SHH is a novel niche factor that induces the vascular repair properties of WJ-MSC and that it may be used in the field of regenerative medicine to enhance vascular repair.

## Methods

All procedures performed comply with Chilean legislation and were approved by the Institutional and Bioethical Use Committee (Faculty of Sciences, University of Chile). UC samples were generously donated by VidaCel S.A. as part of a collaboration.

### WJ-MSC isolation and culture procedures

UC samples from full-term normal pregnancies were used, with informed consent from donor women. WJ-MSC were isolated and characterized as previously described with minor modifications [15]. Briefly, UCs were stored and transported in Dulbecco’s modified Eagle’s medium (DMEM; Thermo Scientific) from maternity facilities to our laboratory and were processed within 24 h postdelivery. Tissues were cut into 2 mm^2^ pieces and blood vessels were discarded. The tissues were digested with collagenase I (1 μg/μL; Thermo Scientific) in phosphate-buffered saline buffer (PBS; pH 7.4) with gentle agitation at 37 °C for 16 h. The resulting cell suspension was subsequently diluted and washed with PBS, and centrifuged to obtain a clean cellular pellet. Cells were seeded in DMEM supplemented with 10% fetal bovine serum (FBS; Biological Industries) and antibiotics (100 U/mL penicillin/Sstreptomycin; Thermo Scientific). WJ-MSC cultures were maintained in a humidified atmosphere containing 5% CO_2_ at 37 °C; after 24 h, nonadherent cells were discarded. Culture medium was frequently changed until cells could be subcultured using 0.05% trypsin-EDTA (Thermo Scientific) up to passages four through six. Because MSC are a heterogeneous stem cell population, we utilized the ISCT (International Society of Cell Therapy) [48] guidelines to characterize them as primary cultures of WJ-MSC (data not shown). We previously showed that WJ-MSC indeed have these characteristics and can be considered MSC [15]. Primary cultures of human umbilical vein endothelial cells (HUVEC) were obtained from full-term normal UC as described previously [49]. Human adipose tissue-derived mesenchymal stem cells (AD-MSC) were cultivated following the protocol of WJ-MSC cultivation.

### Immunofluorescence and immunohistochemistry staining

UC tissues were abundantly washed with PBS to eliminate remaining blood from the umbilical vessels. Segments (1–2 cm) were fixed with 4% paraformaldehyde for 18–24 h in constant agitation at room temperature. After three washes with PBS, the tissue was dehydrated, cleared with Neoclear (Merck Millipore), and embedded in paraffin (Merck Millipore) to subsequently cut with a microtome to generate 20-μm histological sections. For staining, sections were deparaffinized, treated first with a citrate solution (pH 3–3.5), subsequently with 3% H_2_O_2_, and finally with 50% methanol in PBS. Sections were blocked with 5% horse serum in PBS; antibodies were incubated in the same solution overnight at 4 °C (rabbit anti-SHH dilution 1/50; sc-9024; Santa Cruz). After washing with PBS, the secondary antibody was incubated in the blocking solution for 1 h; 4’,6-diamidino-2-phenylindole (DAPI; Sigma) was used for nuclear visualization. Samples were mounted with FluorSave (Merck Millipore) for indirect fluorescence microscopy analysis. Immunohistochemistry staining was performed as previously described [15] using the antibody rabbit anti-SHH.

For primary cell staining, WJ-MSC were seeded onto glass coverslips in DMEM with 10% FBS until 80% confluence. Cells were washed, fixed with 4% paraformaldehyde, blocked and permeabilized with 5% bovine serum albumin (BSA)-0.1% Tween-20. The primary antibody was incubated in the blocking solution overnight at 4 °C (rabbit anti-SHH dilution 1/50; sc-9024; Santa Cruz). For double immunofluorescence staining nonpermeabilized cells were incubated in rabbit anti-SHH and goat anti-PTCH1 (dilution 1/50; sc-6149; Santa Cruz). The secondary antibody was incubated with DAPI for nuclear staining, as well as with phalloidin (0.1 μg/mL). Samples were mounted with FluorSave (Merck Millipore) for indirect fluorescence microscopy.

### RT-PCR and qPCR analysis

mRNA was extracted using E.Z.N.A.® Total RNA Kit I (Omega Biotek), according to the manufacturer’s instructions; 2 μg of RNA were used as the starting material for the retro-transcription. After treating the RNA with DNAse (Promega), cDNA was generated with RT Minus enzyme (200 U/μL; Thermo Scientific), Random Primers (Promega), and 2 mM dNTPs (Thermo Scientific). cDNA was stored at –20 °C until further analysis.

The expression of the SHH pathway components was evaluated via RT-PCR using the primers listed in Additional file 1: Table S1. Relative expression patterns of *PTCH1*, *GLI1*, and *ANGPT1* were quantified with qRT-PCR, relative to *GAPDH* as a housekeeping gene. PCR reactions were carried out using Brilliant II SYBR Green qPCR Master Mix (Stratagene) according to the manufacturer’s instructions and were amplified with qPCR System 3000X (Stratagene). Cycle thresholds (Ct) were generated and analyzed with MxPro Software using the expression ΔΔCt for fold change in gene expression [51, 52].

### Western blot assays

Protein lysates were obtained from WJ-MSC monolayers and homogenized in lysis buffer composed of a 1× protease inhibitor mix (Thermo Scientific). Protein concentration was determined (DC™ Protein Assay; BioRad), and a 50-μg protein concentration was loaded for SDS-PAGE and blotted on 0.45-μm pore nitrocellulose membranes. Membranes were blocked and incubated with anti-SHH or vascular endothelial growth factor (VEGF) antibodies. SHH western blots were carried out as previously described [53] using a 5E1 antibody (Hybridoma supernatant concentrated from Hybridoma Bank; dilution 1/1000). VEGF was detected using rabbit anti-VEGF (Abcam; ab46154; 1/1000). Different positive control samples were used for both proteins (see Results section). Antigens were detected via chemiluminescence using ECL solutions (SuperSignal™ West Pico or Femto Maximum Sensitivity Substrate; Thermo Scientific). Exposed X-ray films (Fujifilm) were analyzed with the Relative Pixel Intensity tool from ImageJ (NIH, USA).

### Pharmacological treatments and conditioned medium (CM) collection

All pharmacological treatments were performed in the absence of serum since FBS contains growth factors that could mask those present in the CM. To evaluate the response of MSC (AD-MSC and WJ-MSC) to SHH pathway stimulation, serum-starved cells were treated for 24 or 48 h with either the SMO agonist Purmorphamine (Pur; 10 μM, DMSO as vehicle; Calbiochem) or recombinant N-Shh (3.3 ng/mL; R&D Systems). 5E1 (5 μg/mL, denaturated antibody as control; Hybridome Bank), a monoclonal antibody that recognizes the epitope that impairs the SHH protein from binding to PTCH1, was used for SHH pathway inhibition.

To evaluate the pro-angiogenic response of WJ-MSC to Pur and 5E1, the pharmacological treatments were dissolved in DMEM 1× (serum free). WJ-MSC were seeded in DMEM with 10% FBS until 80% confluence, washed with PBS, treated for 6–48 h, and lysed for RNA isolation. CM was collected from serum-starved (6–48 h) WJ-MSC cultures grown to 80–90% confluence, immediately frozen in liquid nitrogen, and stored at –80 °C until further use. Importantly, neither Pur nor 5E1 treatments significantly affected the metabolic activity of WJ-MSC as shown via 3-(4,5-dimethyl-2-thiazolyl)-2,5-diphenyl-2H-tetrazolium bromide (MTT) assay (Additional file 2: Figure S1).

### MTT assay

Cells were seeded (1 × 10^4^ cells/well) in 24-well plates with DMEM and 10% FBS. After 24 h, the medium was replaced with 10% DMEM, DMEM, DMEM + Pur (10 μM), or DMEM + 5E1 (5 μg/mL) for 48 h. The MTT reagent (Thermo Scientific) was added (0.5 mg/mL) to evaluate mitochondrial activity. Formazan blue formation was quantified by absorbance at 550 nm.

### Alkaline phosphatase (AP) reporter assay

C3H10T1/2 mesenchymal murine cells were used as reporters of SHH pathway activity because they differentiate into the osteogenic lineage when exposed to the SHH ligand. This can be detected as their AP activity is increased and quantified [54]. C3H10T1/2 were seeded in 0.5% FBS for 24 h and treated for 2 days with fresh WJ-MSC CM (conditioned for 48 h). Afterwards, AP activity was determined using NBT/BCIP (Roche) which stains AP-positive cells with an intense purple color. We used nuclear fast red (NFR) as a nuclear counterstain. Differentiation percentage was determined by the following equation: differentiation percentage = (AP^+^ cells/NFR cells) × 100%. We used at least three independent CM. We used two well-known SHH pathway inhibitors: cyclopamine (Cyc, Infinity, a Smo antagonist) and 5E1 (Hybridome Bank). When using Cyc, reporter cells were pretreated with the inhibitor (10 μM) for 1 h before CM application at 37 °C; we used ethanol, the Cyc vehicle, as a negative control. When using 5E1, the CM was pretreated with the antibody (5 μg/mL) for 1 h at 37 °C and cells were subsequently exposed to 5E1-CM. We used denatured 5E1 (d5E1) by heating it to 95 °C for 5 min as a negative control. We used recombinant N-Shh (3.3 μg/mL; R&D Systems) and Pur (10 μM; Calbiochem) as positive controls.

### Proteome profiler array studies

CM was harvested after culturing cells with serum starvation for 48 h. To analyze the expression of different angiogenic factors of WJ-MSC, 1 mL of CM was assayed using a human angiogenesis array kit (catalog no. ARY007; R&D Systems) according to the manufacturer’s instructions. Spots were detected by enhanced chemiluminescence and quantified by densitometry using the software ImageJ.

### Tubule formation assay

To assess the angiogenic potential of CM from WJ-MSC, we performed a tubule formation assay using HUVEC as previously described [14] with minor modifications. Briefly, HUVEC primary cultures (subcultures 1–4) were serum starved overnight before the assay. Cells were then seeded over solid growth factor-reduced Matrigel (BD Biosciences) in 96-well plates with 24- or 48-h harvested CM. Pur-treated CM was collected at the same time points. We used DMEM as a negative control and EGM-2 (Endothelial Cell Growth Medium; Lonza) as a positive control. After 3–4 h incubation, five photographs were taken per well. Tubular networks were quantified by counting the number of branching points and new tubules formed using ImageJ.

### Chicken CAM assay

For an in vivo evaluation of the angiogenic inductive potential of WJ-MSC, we performed a CAM assay [55]. Fertilized chicken eggs (Rock iso, Agricola Chorombo, Chile) were incubated at 38.5 °C with 75% humidity. At embryonic day 3 (E3), eggs were cleaned with 70% ethanol, and 3 mL of albumin was extracted from each egg; these were subsequently returned to the incubator. On E4, a 2-cm^2^ window was created and an antibiotic solution (penicillin/streptomycin 250 μL, 100 U, 100 mg; Thermo Scientific) was applied prior to reincubation; 24 h before the assay, WJ-MSC (5 × 10^5^ cells) were seeded on Integra® Matrix (diameter = 6 mm) scaffolds with different experimental conditions: DMEM, DMEM + N-Shh (3.3 ng/mL; R&D Systems), or DMEM + Cyc (10 ng/mL; Infinity). On E8, the treated scaffolds were placed on top of the CAM. Integra® Matrices charged with fibroblast growth factor (FGF)2 (20 ng/mL) or DMEM alone were used as positive and negative controls, respectively. On E10, the respective Integra® Matrix scaffolds were reloaded in order to ensure their effect on WJ-MSC. On day E12, we photographed the CAM with a digital camera (HD IC80; Leica, Germany) for quantification. To do this we enhanced the visualization of the blood vessels by injecting cosmetic white facial cream under the CAM and we counted the number of vessels that entered into the scaffold to determine the angiogenic score using the ImageJ software.

### Statistical analysis

At least three independent experiments were carried out for each assay. Values are mean ± SEM, *n* indicates the number of independent cell cultures isolated from different donors. Student’s unpaired *t* tests and analysis of variance (ANOVA) were used to make comparisons between two and more than two groups, respectively. The software Graphpad Prism 5.0b (GraphPad Software Inc., San Diego, CA, USA) was used for data analysis. Results at *P* < 0.05 were considered statistically significant.

## Results

### WJ-MSC are responsive to SHH pathway stimulation

SHH is a growth factor involved in tissue regeneration and angiogenesis throughout postnatal life. Therefore, identifying cellular mediators of SHH signaling during angiogenesis might improve repair processes [37, 56–58]. The UC is a widely used source of stem cells in regenerative medicine treatment [50, 59, 60], and WJ-MSC is a cell population with high angiogenic potential (compared to bone marrow-derived MSC and AD-MSC, among others) [15, 61, 62]. Thus, we evaluated whether the SHH pathway is active in WJ-MSC and whether the SHH ligand regulates the angiogenic induction potential of WJ-MSC through the secretion of angiogenic growth factors as previously reported [14, 15].

First, we characterized the expression of SHH pathway components in WJ-MSC. PTCH1 was detected in the WJ of the umbilical cord (Additional file 3: Figure S2A and B), as well as in cell lysates from WJ-MSC primary cultures (Additional file 3: Figure S2C). We additionally detected mRNA of *SMO*, *GLI1*, *GLI2*, and *GLI3*, other fundamental components of the SHH pathway (Additional file 3: Figure S2D). Overall, the expression of these proteins suggests that the SHH pathway is functionally active in WJ-MSC.

**Fig. 1:**
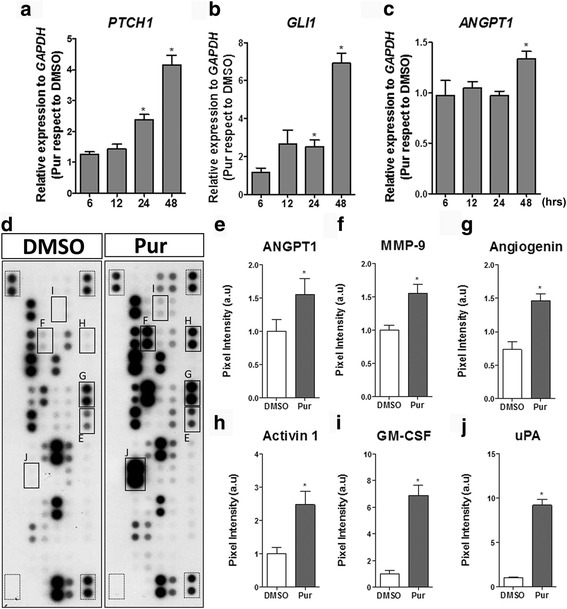
WJ-MSC are responsive to SHH pathway modulation increasing their angiogenic secretome. WJ-MSC were stimulated with purmorphamine (*Pur*) or DMSO (vehicle) in serum absence for the time points indicated, and transcript levels of (**a**) *PTCH1*, (**b**) *GLI1*, canonical SHH target genes, and (**c**) *ANGPT1*, a well described angiogenic factor, were determined by qPCR using *GAPDH* as a normalizing gene. The increment in gene expression is directly indicative of positive pathway signaling. *P < 0.05, one-way ANOVA for three independent umbilical cord samples. To determine the effect of SHH pathway activation in WJ-MSC, conditioned medium (CM) was obtained after 48 h of Pur stimulation and the presence of angiogenic factors was determined by Proteome Profiler Array. **d** Representative membranes showing CM from DMSO and Pur-stimulated WJ-MSC; *squares* indicate molecules in which quantifications are depicted (*dotted squares* indicate internal positive and negative controls). The SHH pathway regulates the secretion of (**e**) angiopoietin 1 (*ANGPT1*), (**f**) matrix metallopeptidase-9 (*MMP-9*), (**g**) angiogenin, (**h**) activin A, (**i**) granulocyte-macrophage colony-stimulating factor (*GM-CSF*), and (**j**) urokinase-type plasminogen activator (*uPA*). *P < 0.05, unpaired Student’s *t* test; CM were obtained from four different WJ-MSC samples

We further explored the activity of the SHH pathway by using a pharmacological activator of the pathway, Pur. qRT-PCR assays demonstrated that levels of *PTCH1* and *GLI1* increased significantly after treating the cells with Pur (Fig. 1a and b). Angiopoietin 1 (*ANGPT1*), a classic angiogenic factor and SHH target [38], also responded to Pur stimulation with a maximum response at 48 h (Fig. 1c). These results provide evidence that WJ-MSC are responsive to the SHH pathway. Using the same methodology, we compared the cellular response of WJ-MSC to that of AD-MSC, another source of MSC broadly studied in the field of regenerative medicine. Relative expression levels of *PTCH1* and *GLI1* (Additional file 4: Figure S3A and B) reveal that AD-MSC are not as responsive to the pathway as are WJ-MSC. In fact, *ANGPT1* levels did not increase after the treatment in AD-MSC (Additional file 4: Figure S3C). We have previously shown that WJ-MSC possess a higher angiogenic potential than AD-MSC [15], and we now demonstrate that these angiogenic properties can be enhanced through SHH pathway modulation. Interestingly, when we compared basal levels of *PTCH1*, *GLI1*, and *ANGPT1* in WJ-MSC and AD-MSC, we found that these transcript levels were lower in WJ-MSC with respect to AD-MSC (Additional file 4: Figure S3D). The latter suggests that the responsiveness to SHH pathway activation (either Pur or N-Shh treatment) is higher in WJ-MSC when compared to AD-MSC.

The positive response of *ANGPT1* in WJ-MSC prompted us to examine whether the SHH pathway can command other angiogenic factors in a global manner. To address this, we analyzed the protein levels of several growth factors present in WJ-MSC CM generated after treatment with Pur for 48 h; the time point was chosen in accord with the highest *ANGPT1* transcriptional level detected (Fig. 1c; Additional file 4: Figure S3C). We confirmed via a secretome array analysis increased levels of ANGPT1 and matrix metallopeptidase (MMP)-9 (Fig. 1d–f), both previously described as SHH targets in other cells types [63, 64]. Interestingly, we also detected angiogenin, a potent angiogenic factor (Fig. 1g). Thus, we consider this angiogenin to be a novel angiogenic SHH target. Moreover, activin A, extensively described in wound healing, and granulocyte-macrophage colony-stimulating factor (GM-CSF) as part of an inflammatory response (Fig. 1h and i), were other SHH targets [65, 66]. Finally, urokinase-type plasminogen activator (uPA; Fig. 1j), an angiogenesis promoter implicated in plasminogen to plasmin activation, was also identified as a SHH target. The latter results suggest that SHH has a potential role in tissue repair.

VEGF is a classic angiogenic factor, but there is conflicting evidence regarding its regulation by canonical SHH signaling [38, 64]. In our experimental settings, SHH did not stimulate VEGF secretion in the presence of Pur (48 h) (Additional file 5: Figure S4A–C). However VEGF has been reported to respond to the SHH pathway in a GLI1-independent manner and in a different time frame [38]. Accordingly, we obtained cell lysates of WJ-MSC stimulated with Pur for 6–48 h and determined VEGF protein levels, but did not observe modulation at any time point. As a positive control of VEGF induction in WJ-MSC, cells were exposed to hypoxia (2% O_2_) for 24 h. Importantly, it was only under this setting that we observed increased VEGF secretion (Additional file 5: Figure S4D and E), suggesting that VEGF is regulated in WJ-MSC but independent of SHH pathway modulation.

It should be noted that a high number of molecules are stimulated by Pur in this proteome array (Fig. 1d). Considering that there is high donor variability in WJ-MSC cultures [14], we only report factors as being SHH targets when they increase significantly in at least three independent UC samples.

### The SHH pathway is pro-angiogenic for WJ-MSC

Having demonstrated that SHH enhances expression and secretion of classical angiogenic molecules in WJ-MSC, we sought to explore the contribution of the SHH pathway in this angiogenic inductive potential. To evaluate this, WJ-MSC were treated with Pur for 24 and 48 h and CM was collected.

**Fig. 2:**
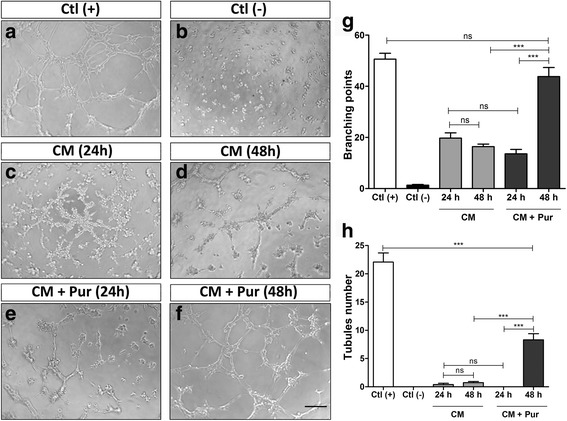
The SHH pathway enhances angiogenic potential of WJ-MSC in vitro. Serum-starved HUVEC, seeded in Matrigel, were treated with conditioned medium (*CM*) from nonstimulated WJ-MSC or with CM from purmorphamine (*Pur*)-stimulated WJ-MSC (CM + Pur) at the indicated time points. EGM-2 was used as positive control (*Ctl +*) and DMEM as negative control (*Ctl -*). The angiogenic response was assessed by counting the number of branches and tubules formed by endothelial cells after 3–4 h. **a**–**f** Representative images of different treatments. **g**, **h** Histograms comparing the number of branching points and tubules between control and experimental conditions as indicated. While there is no difference in these parameters between CM obtained after 24 and 48 h under standard conditions, a strong response was observed after 48 h CM + Pur treatment (CM obtained from 48-h Pur-treated WJ-MSC) which indicates that SHH pathway stimulation enhances the angiogenic capacity of WJ-MSC. CM and CM + Pur were obtained from three independent WJ-MSC cultures. ***P < 0.0001, one-way ANOVA. *Scale bar* = 100 μm. *ns* not significant

We analyzed the effect of the CM in a tubule formation assay using HUVEC primary cultures. HUVEC were treated with EGM-2 (Fig. 2a) to quantify branching points and tubule formation, and treated with DMEM as a negative control (Fig. 2b). In line with our previous results [15], we confirmed that WJ-MSC secrete pro-angiogenic growth factors that stimulate capillary-like structure formation in vitro (Fig. 2c and d); there was no significant difference between using CM collected after 24 or 48 h. Treating the cells for 24 h with CM + Pur displayed similar angiogenic inductive potential in comparison to treating them with CM alone. This suggests that the transcriptional activity of the SHH pathway had not yet significantly influenced the secretion of the growth factor secretion (Fig. 2e). However, we observed a significant inductive angiogenic potential when cells were treated for 48 h with CM + Pur, quantified as an increase in both branching points and tubule formation (Fig. 2f–h). These results indicate that the WJ-MSC secretome not only has a potent angiogenic inductive potential, but can also be enhanced by SHH pathway activation. Therefore, SHH signaling may play a relevant role in the perivascular niche.

### SHH is expressed in the WJ of human umbilical cord

**Fig. 3:**
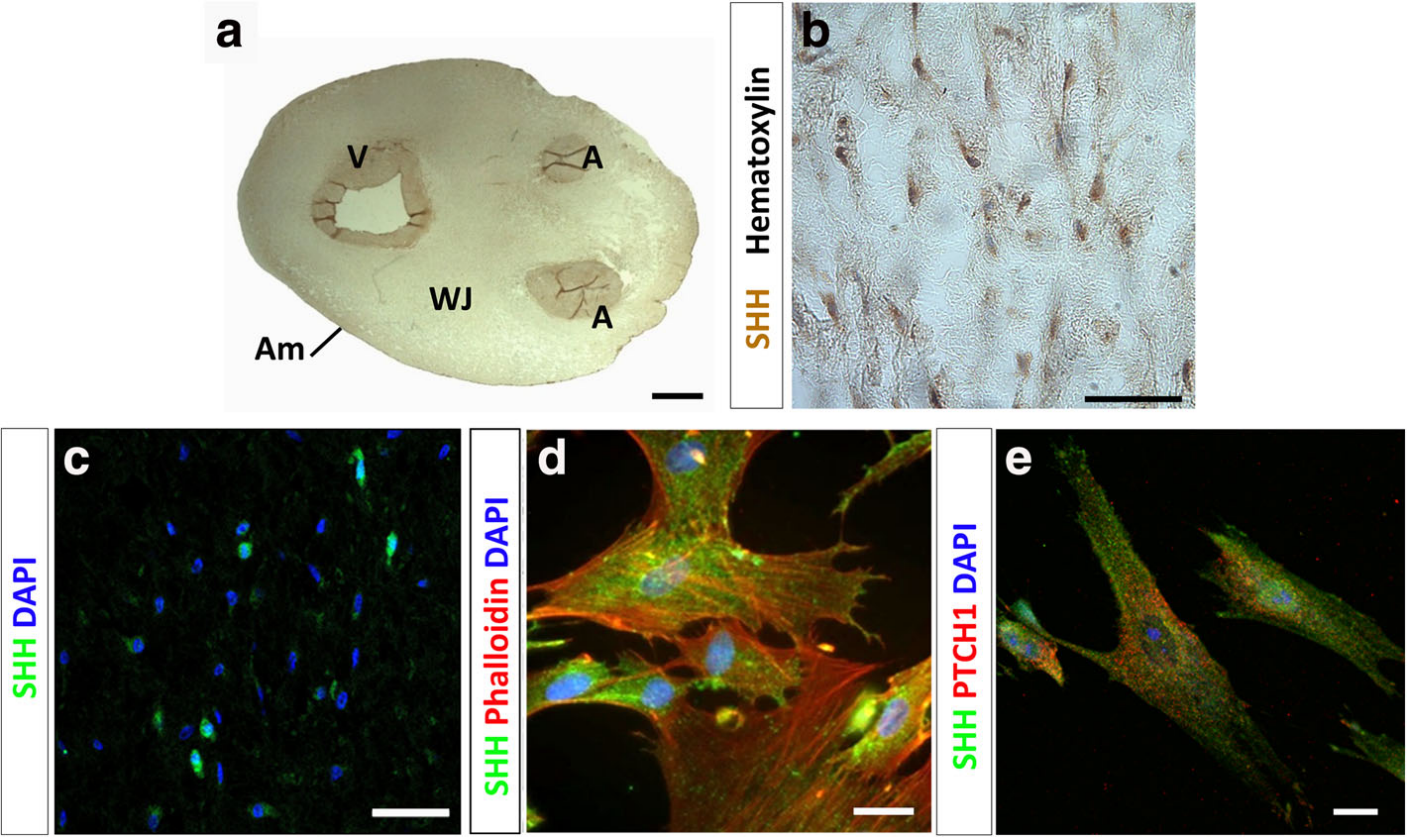
SHH is expressed in human umbilical cord. **a** Representative image of immunohistochemistry against SHH in an umbilical cord section. **b** Imuunohistochemistry shows positive immunoreactivity in cells immersed in the WJ, with hematoxylin staining allowing cell nuclei visualization. **c** SHH^+^ cells are detected in the WJ by immunofluorescence. **d** In primary cultures of WJ-MSC, SHH was detected at the cellular surface. **e** Double immunostaining reveals coexpression of SHH and PTCH1 in WJ-MSC suggesting an autocrine signaling. *Scale bars* = 0.5 cm (**a**), 50 μm (**b**, **c**), and 20 μm (**d**, **e**). *A* artery, *Am* amnios, *DAPI* 4’,6-diamidino-2-phenylindole, *PTCH1* Patched1, *SHH* Sonic hedgehog, *V* vein, *WJ* Wharton’s jelly

SHH is one of the top 15 genes expressed in the UC [46], but little is known about its specific localization and physiological role. Hence, we analyzed SHH expression in the UC through immunohistochemistry and immunofluorescence (Fig. 3). With low magnification, SHH-positive cells can be observed in the vein wall, arteries, and WJ (Fig. 3a). With higher magnification, SHH is detected in stromal cells immersed in the collagen mesh of WJ, which correspond to WJ-MSC (Fig. 3b and c). Interestingly, most but not all WJ-MSC were positive for SHH staining. Since WJ-MSC from the umbilical cord displayed SHH expression, we evaluated its expression in WJ-MSC primary cultures. The positive staining in the cytoplasm (Fig. 3d) suggests a basal activation of the pathway both in vivo and in vitro. Indeed, we detected SHH and PTCH1 coexpression in individual cells (Fig. 3e), suggesting that at least a subpopulation of cells signals in an autocrine fashion. The results evidence that, in WJ, the SHH pathway can act in an autocrine or paracrine fashion within the WJ-MSC population or among different umbilical cell populations.

### WJ-MSC secrete biologically active SHH

**Fig. 4:**
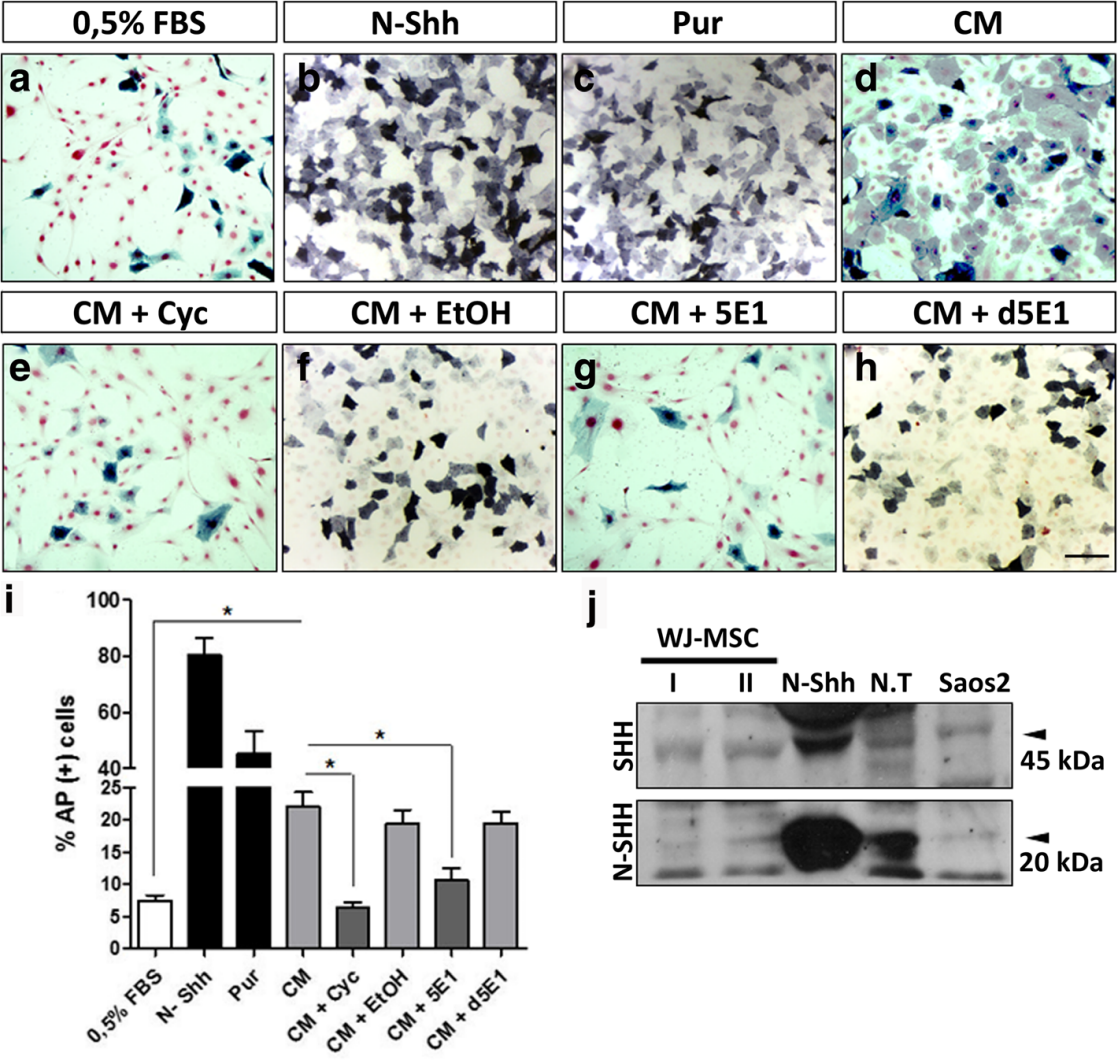
WJ-MSC secrete SHH with biological activity. A reporter assay shows osteogenic differentiation of murine mesenchymal C3H10T1/2 cells in response to WJ-MSC-secreted SHH. AP-positive cells were quantified along with total cells and were graphed. **a** DMEM 0.5% FBS was the CM vehicle. **b**, **c** Pur and recombinant N-Shh were used as positive controls. **d** The CM from three independent WJ-MSC samples were used. To block WJ-MSC-secreted SHH, C3H10T1/2 were preincubated with Cyc before treatment with WJ-MSC CM (**e**) or ethanol as vehicle (**f**). Additionally, the CM was preincubated with 5E1 (**g**) or its denaturated form as control (*d5E1*) (**h**). **i** Histogram quantification is representative for three independent WJ-MSC samples. *P < 0.05, two-way ANOVA. **j** SHH was detected in cells lysates by Western blot. It should be noted that we detected both the immature (N-SHH, 45 kDa) and processed forms of SHH (20 kDa) indicative of WJ-MSC as a source of this ligand (N-Shh corresponds to the commercial ligand and N.T to the embryonic chicken neural tube, both used as positive controls). *Scale bar* = 100 μm. *AP* alkaline phosphatase, *CM* conditioned medium, *Cyc* cyclopamine, *FBS* fetal bovine serum, *Pur* purmorphamine, *SHH* Sonic hedgehog, *WJ-MSC* Wharton’s jelly-derived mesenchymal stem cells

To discern whether WJ-MSC-secreted SHH can promote autocrine or paracrine signaling, we conducted a reporter assay using the murine cell line C3H10T1/2, which differentiates into the osteogenic lineage in response to exogenous SHH [54]. The presence and activity of SHH in WJ-MSC CM can be indirectly detected and quantified by an alkaline phosphatase (AP) activity assay. As expected, lower AP activity was observed with 0.5% FBS (Fig. 4a) and both positive controls induced the osteogenic differentiation of the reporter cells, enhancing AP activity (Fig. 4b and c). Consistent with the above, WJ-MSC CM also significantly increased the number of AP-positive cells (Fig. 4d). Taken together, these results reveal that WJ-MSC secrete functionally active SHH.

To control SHH basal signaling in WJ-MSC, we used two SHH antagonists, Cyc and 5E1. C3H10T1/2 treated with CM + Cyc (Fig. 4e) displayed diminished differentiation, comparable to the control treatment with EtOH (Fig. 4f), confirming that WJ-MSC CM-induced osteogenic differentiation depends on SHH signaling activity. We obtained comparable results when using the monoclonal antibody 5E1. This effect was not observed with denatured 5E1 (d5E1), indicating that the response is specific to SHH signaling (Fig. 4g–i).

To further investigate SHH expression in WJ-MSC lysates, we performed Western blotting. We detected SHH protein in two molecular sizes: approximately 45 and 20 kDa. The 45-kDa SHH corresponds to the full-length SHH precursor, before SHH maturation. The 20-kDa protein corresponds to the processed and lipid-modified N-terminal fragment, the active ligand in the SHH signal transduction pathway (Fig. 4j). *SHH* transcript was also detected in WJ-MSC cell lysates (data not shown). Together, these results indicate that WJ-MSC are a source of SHH and support our hypothesis that there is basal SHH signaling in WJ-MSC.

### WJ-MSC display a basal SHH pathway in vitro

**Fig. 5:**
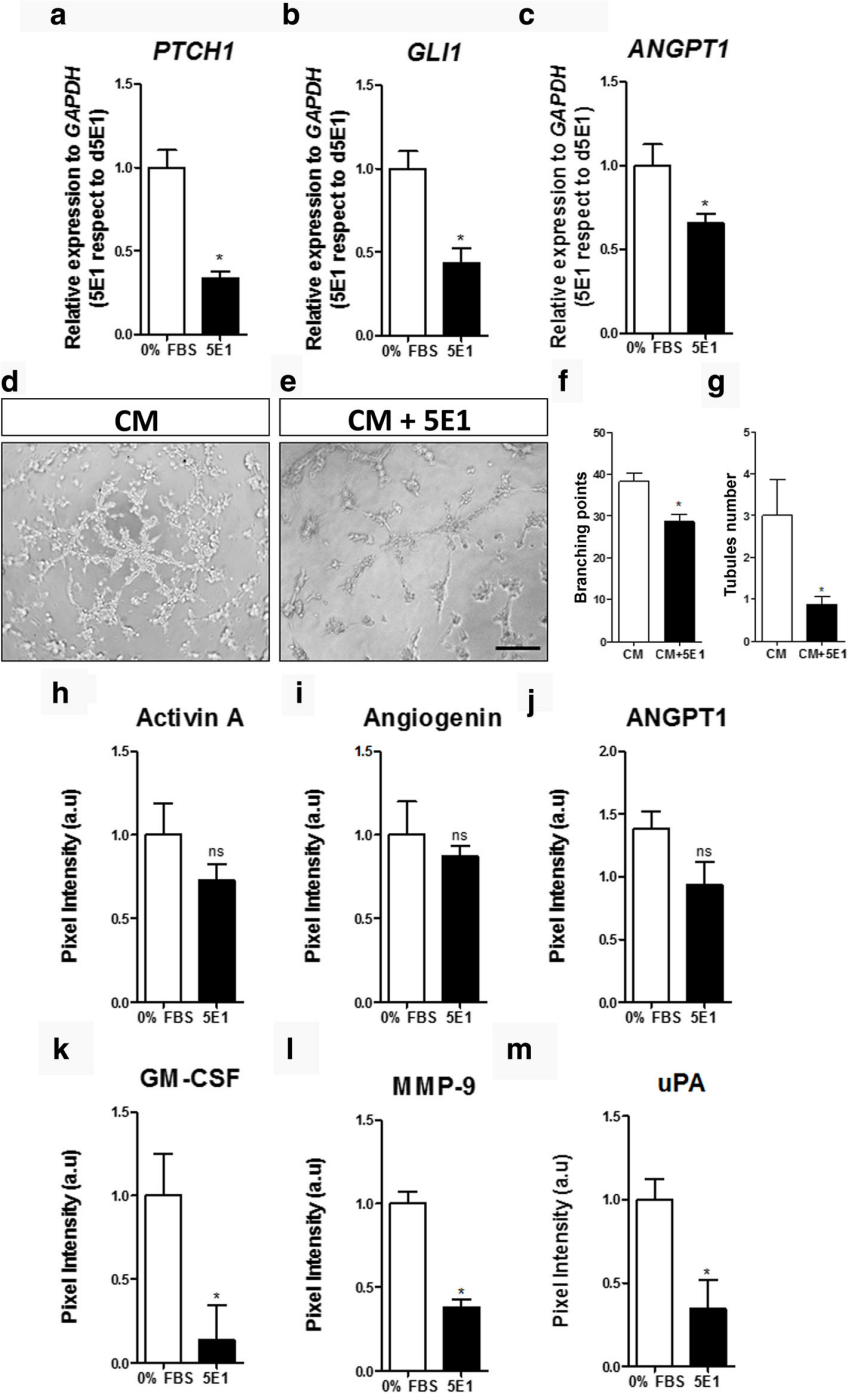
Basal activation of SHH pathway is partially responsible for the angiogenic properties of WJ-MSC. 5E1 was used in the absence of serum to evaluate the contribution of autocrine/paracrine-secreted SHH. After 48 h of treatment, gene expression was evaluated using *GAPDH* as a normalizing gene and 0% fetal bovine serum (*FBS*) as control. Inhibition of the SHH pathway induced a significant decrease in the levels of (**a**) *PTCH1*, (**b**) *GLI1*, and (**c**) *ANGPT1* in three independent WJ-MSC samples. From the same cell cultures, conditioned medium (*CM*) was generated after 48 h in presence of 5E1 and the angiogenic potential was challenged in a tubule formation assay. HUVEC were stimulated with control CM (**d**) or CM from WJ-MSC treated with 5E1 (CM + 5E1) (**e**). Quantification of (**f**) branching points and (**g**) tubules showed that the presence of 5E1 in WJ-MSC cultures induced a decrease in the angiogenic potential. **h**–**m** To decode this effect we analyzed the effect of 5E1 on the secretion of angiogenic factors via proteome analysis. We only observed a significant diminished angiogenic factor secretion for (**k**) granulocyte-macrophage colony-stimulating factor (*GM-CSF*), (**l**) matrix metallopeptidase-9 (*MMP-9*), and (**m**) urokinase-type plasminogen activator (*uPA*). *P < 0.05, unpaired Student’s *t* test. *ANGPT1* angiopoietin 1, *ns* not significant

WJ-MSC secrete SHH and express the ligand receptor PTCH1 (Additional file 3: Figure S2); nonetheless, we further speculated whether WJ-MSC have basal SHH pathway activity. To test this, SHH pathway activity was downregulated. After a 48-h treatment with 5E1, *PTCH1* and *GLI1* transcript levels decreased significantly with respect to the control condition (Fig. 5a and b); similar results were observed for *ANGPT1* (Fig. 5c). Unfortunately, we were unable to validate these results with Cyc; the alkaloid had an inhibitory effect on these genes only in the presence of serum, contrary to our serum-free experimental setting (Additional file 6: Figure S5).

Having used 5E1 to inhibit basal SHH signaling in WJ-MSC, we collected CM from treated cells to evaluate the resulting angiogenic inductive potential in a tubule formation assay. Consistent with our previous results, we observed a decrease in the angiogenic response in HUVEC (Fig. 5d–g), demonstrating that SHH signaling in WJ-MSC is in part responsible for their angiogenic inductive potential. This diminished response is consistent with a canonical SHH-mediated effect on the secretion of the aforementioned angiogenic factors [67].

When analyzing the WJ-MSC secretome after a 48-h treatment with 5E1, there was no significant difference in the levels of activin A, angiogenin, or ANGPT1 (Fig. 5h–j). On the other hand, a significant decrease in the secretion of GM-CSF, MMP-9, and uPA was observed (Fig. 5k–m). These results highlight the need to consider UC donor variability and suggest that different WJ-MSC samples secrete different levels of SHH. Angiogenin, for example, has been previously reported to have differential expression when sampled from different donors [14].

Altogether, our results thus far are indicative of SHH pathway activity in WJ-MSC, the global biological function of which, among other functions, is to regulate the angiogenic inductive effect of WJ-MSC. This makes the use of WJ-MSC in clinical settings particularly attractive. Hence, we tested whether the angiogenic properties of WJ-MSC can be enhanced by the SHH pathway in vivo.

### The inductive effect of SHH on the angiogenic properties of WJ-MSC is conserved in vivo

We chose the CAM assay, a widely validated angiogenic model [55], to validate our results in vivo. As we have previously shown [15], WJ-MSC maintain their angiogenic properties in a three-dimensional setup such as on an Integra® Matrix (IM; Integra® LifeSciences Corp., Plainsbro, NJ, USA), which acts as a scaffold and supports WJ-MSC in the CAM [68, 69]. WJ-MSC were seeded onto the scaffold and stimulated with recombinant N-Shh (3.3 ng/mL) or Cyc (10 ng/mL) for 24 h. After this pretreatment, WJ-MSC-embedded scaffolds were positioned on top of the CAM to evaluate their angiogenic potential after 4 days. Treatment was repeated in ovo in order to maintain the effect on SHH pathway modulation on WJ-MSC.

**Fig. 6:**
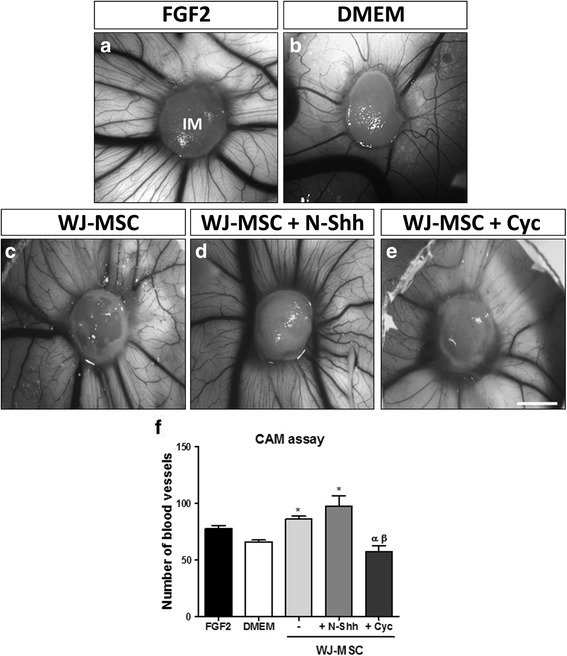
SHH pathway is active in WJ-MSC in vivo and enhances their pro-angiogenic properties. In vitro pretreated Wharton’s jelly-derived mesenchymal stem cells (*WJ-MSC*) (+ N-Shh or Cyc) were applied on top of the chorioallantoic membrane (*CAM*) of chicken embryos. Treatment was repeated after 48 h in order to maintain the effect on SHH pathway modulation in WJ-MSC. The angiogenic response was evaluated after 96 h. Recombinant fibroblast growth factor 2 (*FGF2*), a potent angiogenic stimulator, was used as a positive control (**a**), and (**b**) Dulbecco’s modified Eagle’s medium (*DMEM*) was used as negative control (WJ-MSC vehicle). **c** WJ-MSC seeded in Integra Matrix (*IM*). WJ-MSC seeded in Integra Matrix plus N-Shh (**d**) and Cyc (**e**). **f** Assay quantification. *P < 0.05, one-way ANOVA (WJ-MSC, *n* = 3; chicken eggs, *n* = 9). *Scale bar* = 5 mm. *Cyc* cyclopamine, *SHH* Sonic hedgehog

The FGF2 positive control induced a strong angiogenic response. Interestingly, the angiogenic response was comparable between the FGF2 and DMEM controls. This is likely due to the inductive potential of the scaffold itself (Fig. 6a and b). Representative CAM images depict the parallel pattern of blood vessels that enter the treated area, described as a characteristic “spoke wheel” pattern [70]. WJ-MSC stimulated the vascular network, confirming that the WJ-MSC secretome has an active inductive potential in ovo (Fig. 6c–f). Remarkably, we found that WJ-MSC stimulated with exogenous N-Shh induced angiogenesis in the CAM. In other words, the formation of a significant number of novel vessels indicates that the WJ-MSC angiogenic inductive media (N-Shh) acted in the vascular niche itself (Fig. 6d). In contrast, the Cyc treatment reduced the angiogenic properties that WJ-MSC displayed in vivo (Fig. 6e).

## Discussion

It is well known that MSC have huge potential applications in regenerative medicine. In recent years we have seen the emergence of numerous reports in this field, which is necessary due to the need for new therapeutic alternatives. The wide use of MSC in multiple diseases differs in the consensus about which type of cell or dosage it is better to use [71]. In this context, little is known about the cellular mechanisms that command the therapeutic properties of MSC [3, 72]. WJ-MSC are attractive in this field due to their differentiation potential, highly proliferation rate, and enriched secretome. Over the past years their potential has been widely explored in different areas, using them either as a stem cell population plausible for differentiation [73–75] or as trophic mediators, promoting neural regeneration [62, 76–80], immunomodulation [81–83], wound healing [13, 16, 84, 85], and angiogenesis [14, 15]. Thus, the use WJ-MSC or even their derivatives such as extracellular vesicles [86–88] has increasing academic and clinical interest. The angiogenic properties of WJ-MSC have already been described. Hence, the aim of this study was to reveal cellular mechanisms controlling these properties, and how they can be enhanced for a potential application in medicine.

### SHH autocrine/paracrine signaling in WJ-MSC

MSC isolated from different organs exhibit unique features and it has been suggested that the native tissue environment or embryonic origin imprints such character [89, 90]. SHH pathway signaling is present in multiple niches modulating stem cell maintenance, cell proliferation [91], or differentiation [92–94]. The SHH pathway is also active during UC development [95, 96]; however, its specific contribution to umbilical-derived stromal cell physiology has not been investigated.

As several populations are positive for SHH in the UC, the ligand expression can be relevant for other cells inside umbilical stroma or vessels. Specifically, based on SHH and PTCH1 expression, WJ-MSC secrete SHH and respond to it, which suggests an autocrine/paracrine signaling. Notoriously, the expression of ligand and receptor is heterogeneous among cells and possibly between different donors (data not shown). Furthermore, the use of 5E1 as a blocking antibody allows us to conclude that WJ-MSC display basal SHH pathway activity in vitro as evidenced by *PTCH1* and *GLI1* modulation. Our results suggest that WJ-MSC display SHH autocrine or paracrine signaling as shown already for MSC in other niches [97–100]. However, we must keep in mind that there seems to be a subpopulation of SHH-positive cells within the WJ. Hence, we cannot establish if it is the same subpopulation that secretes and responds to SHH that displays pro-angiogenic capacity or whether a subpopulation of WJ-MSC (not necessarily the same subpopulation) is only responsive to the ligand. It remains to be determined if a particular subpopulation defines the angiogenic potential in every WJ-MSC cell culture, a matter that should be considered for future clinical applications.

The SHH pathway modulates pro-angiogenic secreting capabilities. Interestingly, recent evidence supports the relevance of canonical SHH signaling in MSC-like cells in vascular niches, proposing GLI1 as marker in these specific MSC populations [101, 102]. These evidences highlight the relevance of the SHH pathway in MSC biological functions that have been studied for their clinical application. Certainly, as shown in this work, autocrine and/or paracrine signaling mechanisms suggest SHH involvement in the secretion of growth factors involved in angiogenesis. More research is needed to understand if this relevant signaling pathway also underlies other functions in WJ-MSC, such as cell survival, proliferation, or the inflammatory response.

### SHH is a novel angiogenic factor modulator in WJ-MSC

Pro-angiogenic inductive properties of WJ-MSC have already been described [14, 15], but the underlying mechanism responsible has not been widely addressed. SHH is well known as a morphogen and mitogen during embryonic development [103] and has been proposed as a niche factor in postnatal life [104]. In adults, SHH has been described as a promoter of muscular and cardiac regeneration after ischemic events [21, 37, 56]. In this context, the SHH pro-angiogenic stimulus could be relevant for the stromal response to injury, particularly MSC, which are considered as therapeutic tools in these pathologies [105].

In addition to the basal SHH signaling in WJ-MSC described here, we show that the use of Pur as an external positive activator of the pathway enhances their pro-angiogenic secreting properties. Of note, we used a serum-free experimental design, a condition that has been reported as an inducer of angiogenic factors in bone marrow-derived MSC [106]. Nevertheless, recent evidence indicates that this might not occur in WJ-MSC [107].

Our results show the positive modulation of growth factors previously reported as SHH targets, such as the matrix metallopeptidase MMP-9 and ANGPT1. MMP-9 is a strong angiogenic inductor and was described as a SHH target in a pathological scenario [64, 108, 109] but is also required for migration of endothelial cells. ANGPT1 is another classic angiogenic factor induced by SHH and was found to be upregulated in WJ-MSC, in agreement with previous reports in other cell lines [38, 63, 110]. These results confirm the specificity of the WJ-MSC response to SHH signaling. Moreover, we also detected novel angiogenic targets from SHH pathway in WJ-MSC: angiogenin, activin A, GM-CSF, and uPA.

Angiogenin, which acts on endothelial cells promoting their migration and invasion, additionally impacts positively the activity of other connoted angiogenic factors, such as VEGF, FGF2, aFGF, and EGF [111], all secreted by WJ-MSC. Activin A is member of the transforming growth factor-β (TGF-β) superfamily and its role in angiogenesis is not yet clear. Different reports have shown that activin A acts either as a pro- or anti-angiogenic factor depending on the context. Both activities have been described in endothelial cells; while in the CAM assay this molecule inhibits angiogenesis, it promotes the process in corneal angiogenesis [112]. Additionally, activin A is a promoter of wound healing acting over processes such as re-epithelialization and granulation tissue formation, but also can act as a profibrotic factor [65].

GM-CSF has an angiogenic effect beyond its function in hematopoiesis. This factor acts directly over the endothelium promoting proliferation and migration of endothelial cells [113]. Interestingly, GM-CSF promotes plasminogen activator secretion which, in turn, activates plasmin, also with angiogenic properties besides its main role in fibrinolysis [114]. Plasmin promotes endothelial cell migration by degrading extracellular membrane (ECM) proteins (directly) or by the release of angiogenic growth factors bonded to the ECM (indirectly). As zymogen, plasminogen activation requires the participation of specific activators and inhibitors [115]; among the activators are plasminogen activator (upregulated by GM-CSF) and uPA, both targets of SHH signaling in WJ-MSC, suggesting an important role of plasmin in SHH-induced angiogenesis. uPA also induces the release of growth factors from the ECM for proteolysis, it activates additional proteinases, such as MMP-9 [116], and, after translocation to the nucleus, activates expression of VEGF receptors [117, 118]. SHH appears to regulate plasmin activation as WJ-MSC CM stimulates uPA expression. Accordingly, in mouse brain endothelial cells, in which SHH modulates plasmin activation indirectly although through other components, SHH stimulates tPA expression (tissue plasminogen activator) and represses PAI-1 (plasminogen activator inhibitor) expression [119, 120], promoting in vitro cerebral angiogenesis.

VEGF is the most prominent angiogenic and vasculogenic factor and is generally considered a SHH signaling target, although GBS have not been reported [38]. Interestingly, we found that VEGF is not a SHH signaling target in WJ-MSC. This result is in line with reports indicating that SHH does not stimulate expression of VEGF in HUVEC (located in the same niche as WJ-MSC) or fibroblasts [63, 121]. Still, we must consider the experimental settings that led to this result. Cells were seeded under atmospheric or “normoxic” conditions (21% O_2_), which greatly differs from physiological hypoxia (1.5–8%) [122]. Hypoxia is the major physiological inductor of angiogenesis [123, 124] and VEGF is one of the most prominent factors stimulated by HIF-1α [125]. HIF-1α stimulates SHH expression in fibroblasts and cardiomyoblasts [126, 127] and SMO in pancreatic cancer [128], which suggests that, in situ, hypoxia could also induce SHH signaling in WJ-MSC. The latter in turn suggests that this pathway could be one of the pro-angiogenic downstream effectors of HIF-1α, as has been described in cardiac ischemia [21, 56], also correlating with the reported beneficial effects of SHH signaling in muscle ischemia [21, 37, 129]. Therefore, while VEGF might not be a relevant component of the SHH-induced angiogenic secretome under normoxia, it may be an important target under hypoxia or physiological oxygen levels.

### Possible relevance of SHH signaling in UC tissue

We cannot overlook that SHH expression in the WJ-MSC is a remnant of the active SHH signaling during embryonic development; however, SHH is one of the most highly expressed genes in the UC and some reports have indicated that SHH signaling is active early on but decreases as pregnancy progresses. Stunkel et al. [47] studied gene expression during two stages in advanced pregnancies, and SHH signaling pathway components (*SMO*, *GLI2*, and *GLI3*) were among the most highly expressed early on. Nevertheless, their expression diminished in the advanced group. Analysis of cord blood methylation patterns (i.e., transcription patterns) of multiple genes revealed that *GLI3* displayed decreased methylation, which could correlate with increased expression. Whereas *GLI3* is largely described as a transcriptional repressor of SHH pathway activity, increased expression would relate to blowing activity of SHH. This evidence confirms SHH pathway activity in the UC, which is maintained in WJ-MSC primary cultures.

## Conclusion

WJ-MSC are pro-angiogenic but their complex and diverse secretome is composed of both pro- and anti-angiogenic factors. The presence of these apparent antagonistic factors highlights an important property—angiogenesis is a dynamic process and involves inductive and negative regulatory signals, promoting a controlled process in accordance with a homeostatic scenario and with the participation of multiple cell populations. Still, it remains unclear whether the WJ-MSC secretome can act on other target cells besides endothelial cells. Future research should address if WJ-MSC influence other cell populations, such as perivascular cells or inflammatory cells in vivo.

Summarizing, the presented data provide insight into the pro-angiogenic properties of WJ-MSC and how the SHH signaling pathway acts to mediate this response. Multiple aspects of the extracellular environment might influence the WJ-MSC paracrine activity. Further investigation will be required to establish how the WJ-MSC secretome is regulated by the niche; that is, how exactly can paracrine signaling be modulated spatiotemporally by the microenvironment.

In conclusion, our work positions the SHH pathway as a therapeutic target to be modulated in MSC for regenerative medicine purposes.

## Additional files

Additional file 1: Table S1. RT-PCR and qPCR primers used in this study. All the primers were assayed with Tm = 60 °C. *GAPDH* primers used for RT-PCR and qRT-PCR were the same. (DOC 30 kb)

Additional file 2: Figure S1. SHH pathway modulation does not alter metabolic activity in WJ-MSC. Morphologic appearance of WJ-MSC after 48 h in (A) 10% FBS, (B) 0% FBS, (C) 0% FBS plus Pur, and (D) 0% FBS plus 5E1. (E) WJ-MSC cell number does not change under experimental conditions assayed. (F) MTT assay indicates that there is not a significant decrease in the metabolic activity in WJ-MSC after 48 h under experimental conditions as indicated. *P < 0.05, one-way ANOVA. (TIF 2932 kb)

Additional file 3: Figure S2. WJ-MSC express PTCH1, the SHH receptor, along with other components of the signaling pathway. PTCH1 was found in WJ-MSC immersed in the Wharton jelly (A,B). (C) PTCH1 was detected in cell lysates from primary cultures, along with positive controls (S2: Saos-2; E.C: endothelial cells; N.T: chicken embryonic neural tube). (D) Expression of other main components of the SHH pathway at mRNA level: *SMO*, *GLI1*, *GLI2*, and *GLI3*, validated in three independent samples. (TIF 3677 kb)

Additional file 4: Figure S3. WJ-MSC and AD-MSC respond differentially to SHH pathway stimulation. Cells were treated with Pur or N-Shh, and the response was determined by measuring the levels of (A) *PTCH1*, (B) *GLI1*, and (C) *ANGPT1* by qPCR. WJ-MSC proved to be more responsive to SHH pathway stimulation than AD-MSC. (WJ-MSC *n* = 4; AD-MSC *n* = 5; *P < 0.05 unpaired Student’s *t* test for comparison between AD and WJ-MSC in each treatment). (D) Quantification of basal levels of *PTCH1*, *GLI1*, and *ANGPT1* of WJ-MSC when compared to AD-MSC. Expression levels of the three genes were lower in WJ-MSC cultures (WJ-MSC *n* = 4; AD-MSC *n* = 5). (TIF 1095 kb)

Additional file 5: Figure S4. VEGF is not a target of the SHH pathway in WJ-MSC. (A) VEGF secretion was not stimulated after pathway activation in WJ-MSC, as determined by Proteome Profiler Array. (B) WJ-MSC were stimulated with Pur and cell lysates were obtained after 6, 12, 24,and 48 h; β-actin was used as control. (C) Quantification of (B) showed that there is no significant increase in VEGF levels after pathway activation in four independent samples. (D) WJ-MSC were submitted to hypoxic oxygen levels (2% vs 21%) and HIF-1α was quantified by Western blot in to confirm the hypoxic cellular response. (E) Hypoxia stimulated secretion of VEGF in WJ-MSC after 48 h of treatment. A–E: *P < 0.05 unpaired Student’s *t* test; C,D: *P < 0.05, one-way ANOVA. (TIF 1957 kb)

Additional file 6: Figure S5. Serum dependence of Cyc inhibition in WJ-MSC. Cyc was effective in decreasing *PTCH1* levels only in serum-supplemented medium. (A) In the absence of serum, Cyc (10 μM) did not induce a significant decreased in *PTCH1* levels. (B) In 10% FBS, the standard conditions of WJ-MSC culture, we observed only two time-points with diminished *PTCH1* expression. (C) Lower concentration of the inhibitor still gave a result, but in the presence of serum. *P < 0.05, one-way ANOVA. (TIF 1470 kb)

## Abbreviations

AD-MSC: adipose tissue-derived mesenchymal stem cells
ANGPT1: Angiopoietin 1
AP: Alkaline phosphatase
CAM: Chorioallantoic membrane
CM: Conditioned medium
Cyc: Cyclopamine
DAPI: 4’,6-Diamidino-2-phenylindole
DMEM: Dulbecco’s modified Eagle’s medium
ECM: Extracellular membrane
FBS: Fetal bovine serum
FGF: Fibroblast growth factor
GBS: GLI binding sites
GM-CSF: Granulocyte-macrophage colony-stimulating factor
HUVEC: Human umbilical vein endothelial cells
MMP: Matrix metallopeptidase
MSC: Mesenchymal stem cells
MTT: 3-(4,5-Dimethyl-2-thiazolyl)-2,5-diphenyl-2H-tetrazolium bromide
NFR: Nuclear fast red
PBS: Phosphate-buffered saline
PTCH1: Patched1
Pur: Purmorphamine
SHH: Sonic hedgehog
SMO: Smoothened
UC: Umbilical cord
uPA: Urokinase-type plasminogen activator
VEGF: Vascular endothelial growth factor
WJ-MSC: Wharton’s jelly-derived mesenchymal stem cells
