# Supplementary material for: Sonic hedgehog (SHH) signaling improves the angiogenic potential of Wharton’s jelly-derived mesenchymal stem cells (WJ-MSC)
Source: Stem Cell Res Ther. 2017 Sep 29;8:203. doi: 10.1186/s13287-017-0653-8 (PMC5622478; doi:10.1186/s13287-017-0653-8)
Supplement: Supplementary file 1 — RT-PCR and qPCR primers used in this study. All the primers were assayed with Tm = 60 °C. GAPDH primers used for RT-PCR and qRT-PCR were the same. (DOC 30 kb) [file 13287_2017_653_MOESM1_ESM.doc]

**Additional file 1: Table S1. RT-PCR and qPCR primers used in this study**. All the primers were assayed with Tm = 60°C. *GAPDH* primers used for RT-PCR and qRT-PCR were the same.

|  | **Forward** | **Reverse** |
| --- | --- | --- |
| *SMO* | GTTCTCCATCAAGAGCAACCAC | CGATTCTTGATCTCACAGTCAGG |
| *GLI1* | GGAGAAGCGTGAGCCTGAATC | TGGATGTGCTCGCTGTTGATG |
| *GLI2* | CACCGCTGCTCAAAGAGAA | TCTCCACGCCACTGTCATT |
| *GLI3* | CGAACAGATGTGAGCGAGAA | TTGATCAATGAGGCCCTCTC |
| *PTCH1* | GGTGGAAGTTGGAGGACGAG | CGCTTCTGTGGTCAGGACATTAG |
| *GLI1* | GGAGAAGCGTGAGCCTGAATC | TGGATGTGCTCGCTGTTGATG |
| *ANGPT1* | CAGGAGGATGGTGGTTTGATG | TGGTTTTGTCCCGCAGTATAG |
| *GAPDH* | TGCACCACCAACTGCTTAGC | GGCATGGACTGTGGTCATGAG |
