# Supplementary figures and images for: Sonic hedgehog (SHH) signaling improves the angiogenic potential of Wharton’s jelly-derived mesenchymal stem cells (WJ-MSC)
Source: Stem Cell Res Ther. 2017 Sep 29;8:203. doi: 10.1186/s13287-017-0653-8 (PMC5622478; doi:10.1186/s13287-017-0653-8)

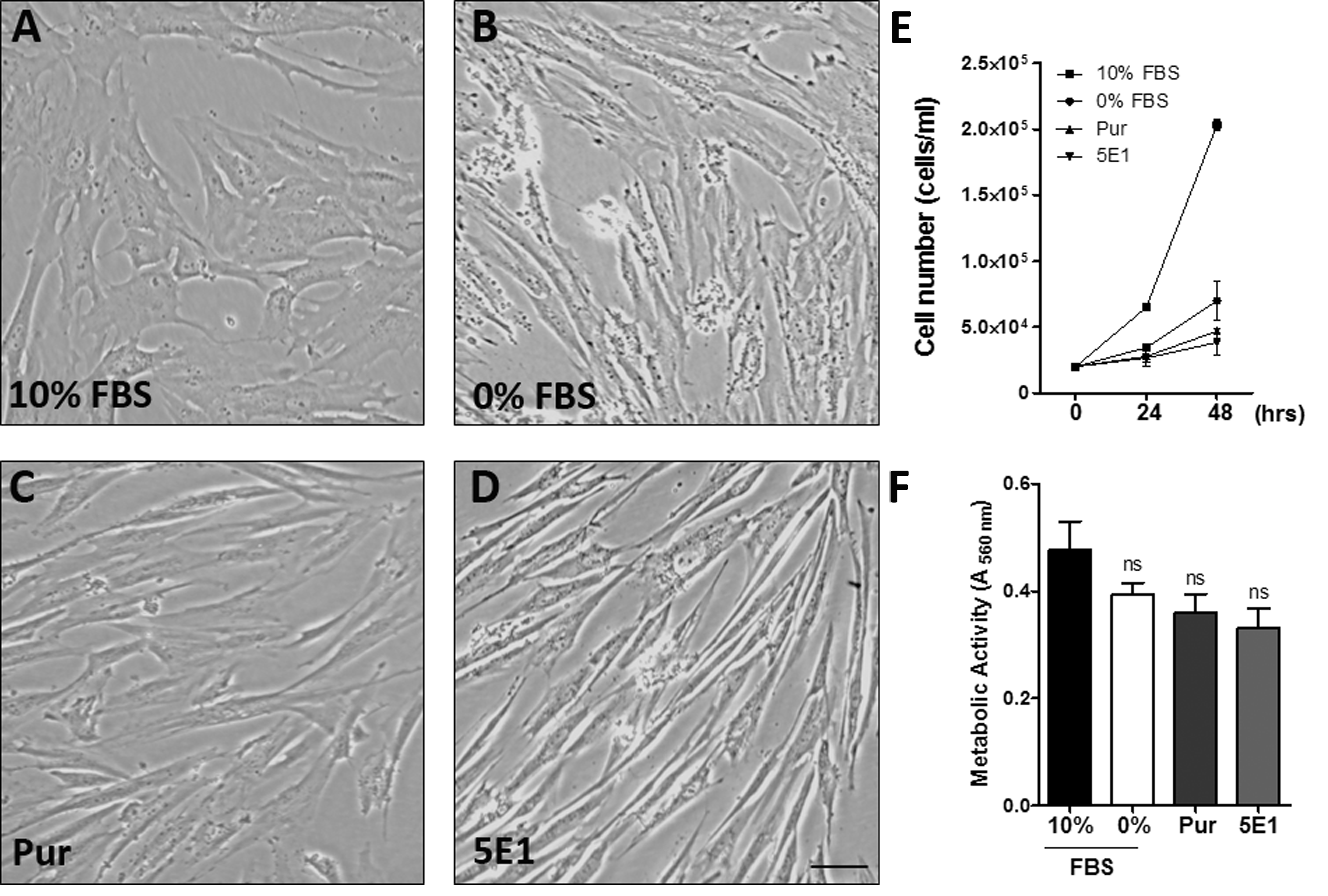

Supplement: Supplementary file 2 — SHH pathway modulation does not alter metabolic activity in WJ-MSC. Morphologic appearance of WJ-MSC after 48 h in (A) 10% FBS, (B) 0% FBS, (C) 0% FBS plus Pur, and (D) 0% FBS plus 5E1. (E) WJ-MSC cell number does not change under experimental conditions assayed. (F) MTT assay indicates that there is not a significant decrease in the metabolic activity in WJ-MSC after 48 h under experimental conditions as indicated. *P < 0.05, one-way ANOVA. (TIF 2932 kb) [file 13287_2017_653_MOESM2_ESM.tif]

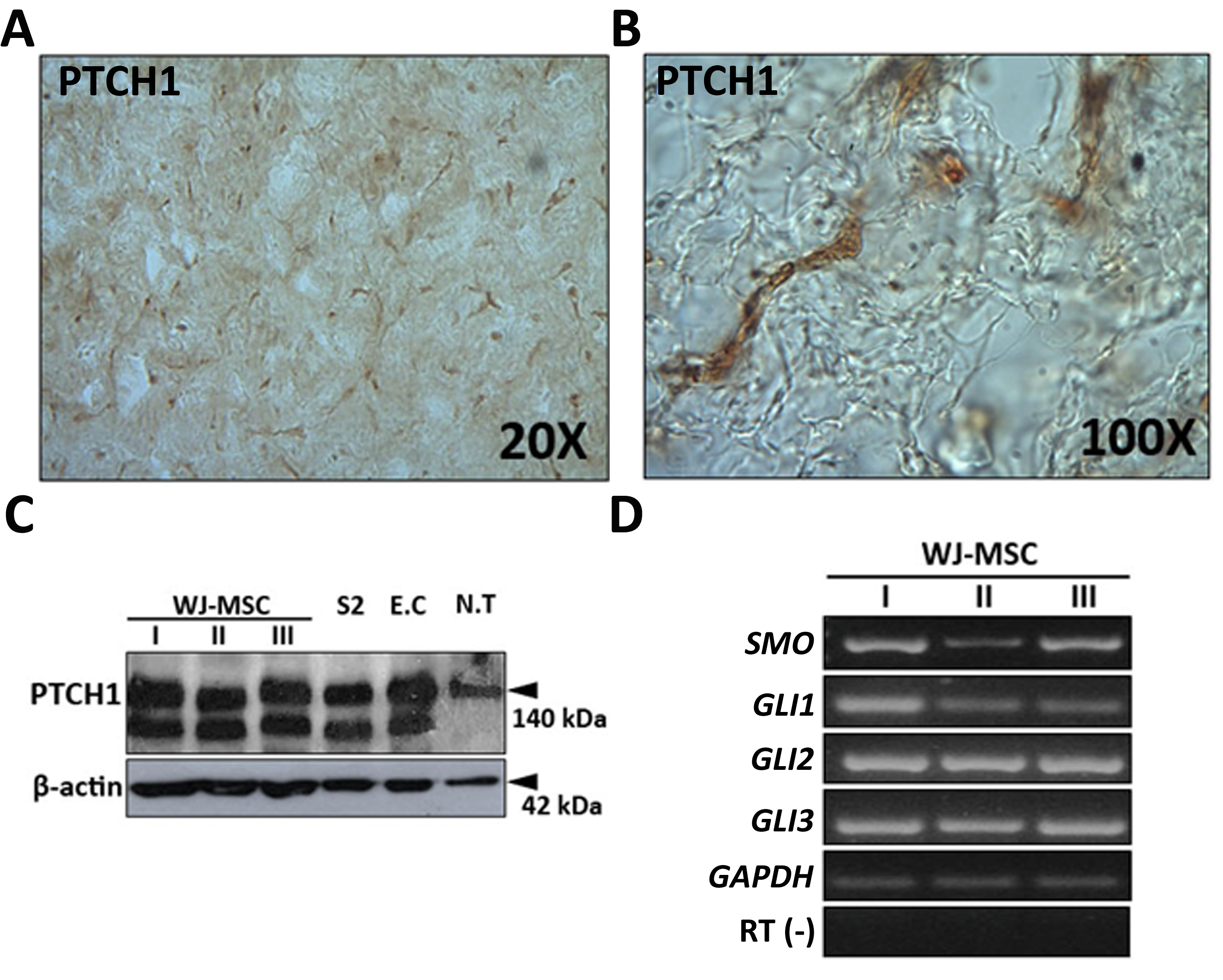

Supplement: Supplementary file 3 — WJ-MSC express PTCH1, the SHH receptor, along with other components of the signaling pathway. PTCH1 was found in WJ-MSC immersed in the Wharton jelly (A,B). (C) PTCH1 was detected in cell lysates from primary cultures, along with positive controls (S2: Saos-2; E.C: endothelial cells; N.T: chicken embryonic neural tube). (D) Expression of other main components of the SHH pathway at mRNA level: SMO, GLI1, GLI2, and GLI3, validated in three independent samples. (TIF 3677 kb) [file 13287_2017_653_MOESM3_ESM.tif]

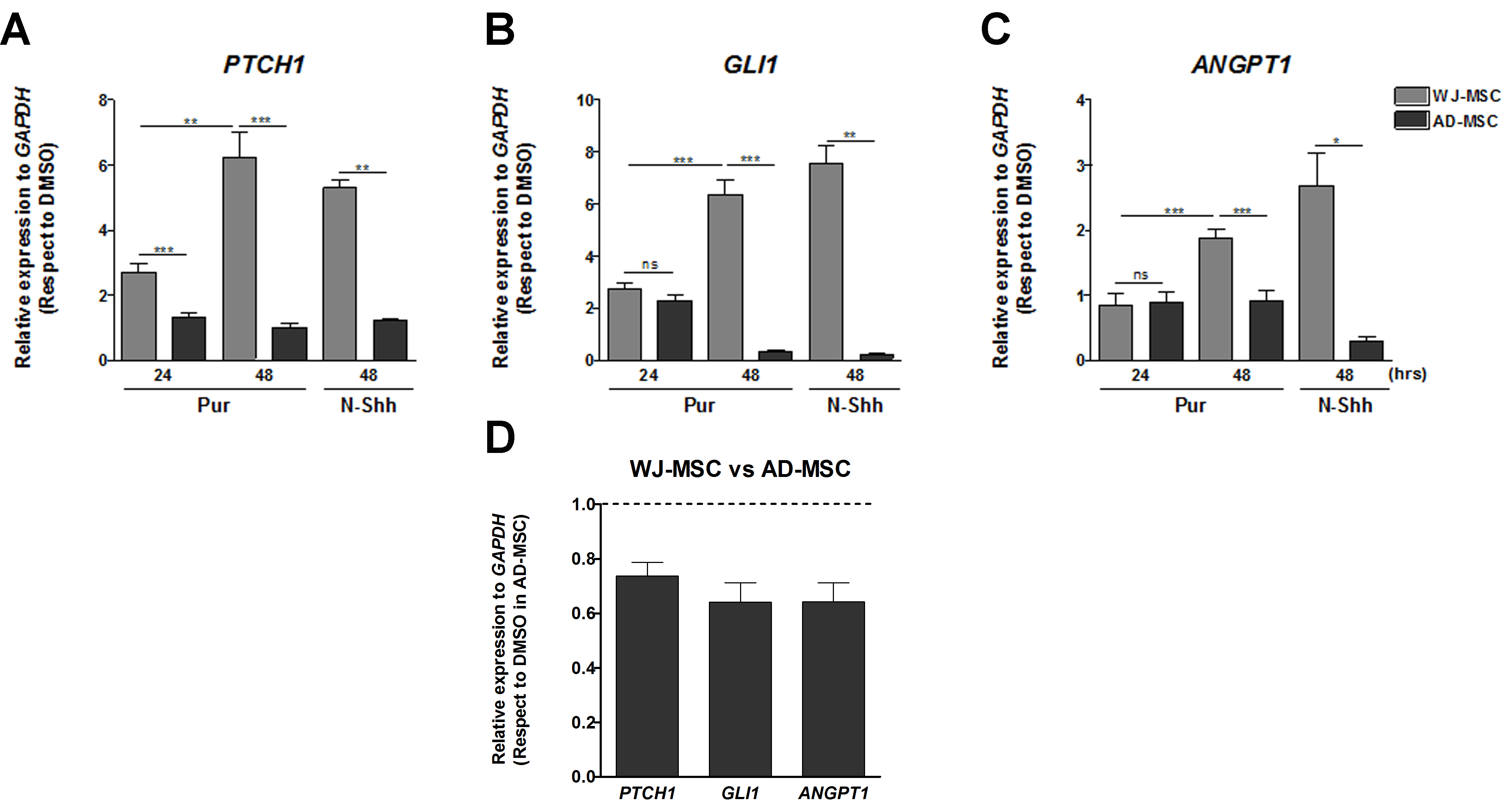

Supplement: Supplementary file 4 — WJ-MSC and AD-MSC respond differentially to SHH pathway stimulation. Cells were treated with Pur or N-Shh, and the response was determined by measuring the levels of (A) PTCH1, (B) GLI1, and (C) ANGPT1 by qPCR. WJ-MSC proved to be more responsive to SHH pathway stimulation than AD-MSC. (WJ-MSC n = 4; AD-MSC n = 5; *P < 0.05 unpaired Student’s t test for comparison between AD and WJ-MSC in each treatment). (D) Quantification of basal levels of PTCH1, GLI1, and ANGPT1 of WJ-MSC when compared to AD-MSC. Expression levels of the three genes were lower in WJ-MSC cultures (WJ-MSC n = 4; AD-MSC n = 5). (TIF 1095 kb) [file 13287_2017_653_MOESM4_ESM.tif]

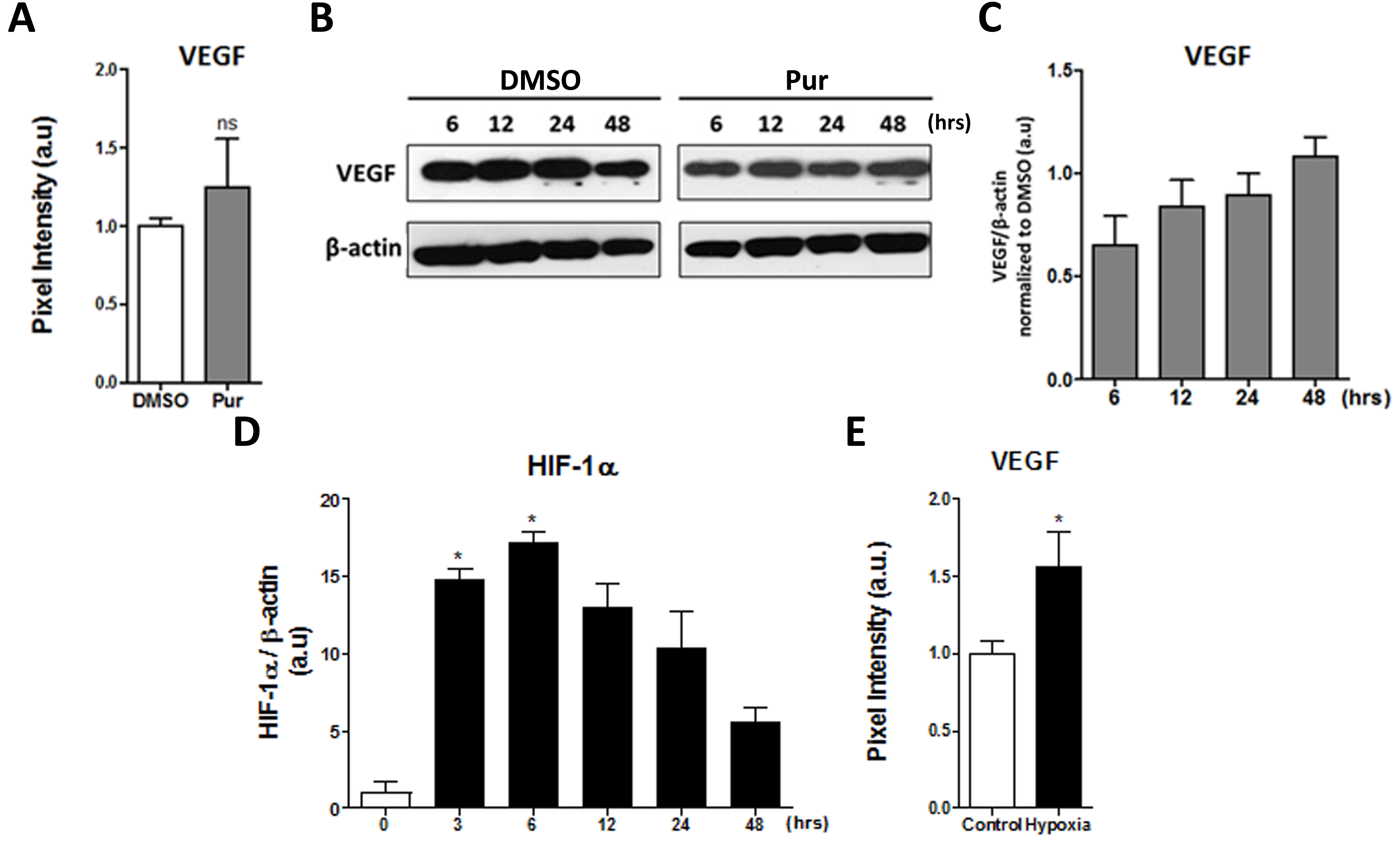

Supplement: Supplementary file 5 — VEGF is not a target of the SHH pathway in WJ-MSC. (A) VEGF secretion was not stimulated after pathway activation in WJ-MSC, as determined by Proteome Profiler Array. (B) WJ-MSC were stimulated with Pur and cell lysates were obtained after 6, 12, 24,and 48 h; β-actin was used as control. (C) Quantification of (B) showed that there is no significant increase in VEGF levels after pathway activation in four independent samples. (D) WJ-MSC were submitted to hypoxic oxygen levels (2% vs 21%) and HIF-1α was quantified by Western blot in to confirm the hypoxic cellular response. (E) Hypoxia stimulated secretion of VEGF in WJ-MSC after 48 h of treatment. A–E: *P < 0.05 unpaired Student’s t test; C,D: *P < 0.05, one-way ANOVA. (TIF 1957 kb) [file 13287_2017_653_MOESM5_ESM.tif]

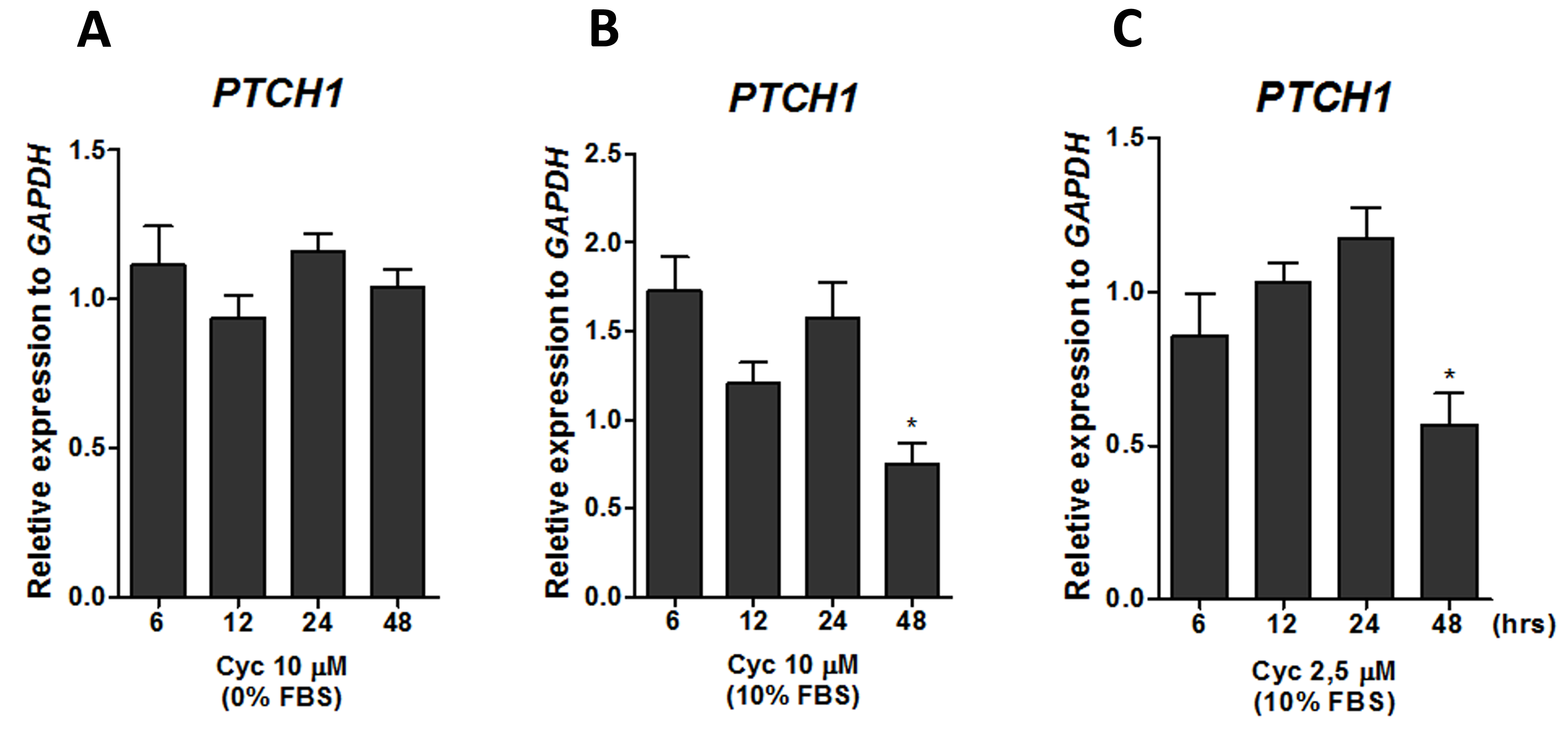

Supplement: Supplementary file 6 — Serum dependence of Cyc inhibition in WJ-MSC. Cyc was effective in decreasing PTCH1 levels only in serum-supplemented medium. (A) In the absence of serum, Cyc (10 μM) did not induce a significant decreased in PTCH1 levels. (B) In 10% FBS, the standard conditions of WJ-MSC culture, we observed only two time-points with diminished PTCH1 expression. (C) Lower concentration of the inhibitor still gave a result, but in the presence of serum. *P < 0.05, one-way ANOVA. (TIF 1470 kb) [file 13287_2017_653_MOESM6_ESM.tif]
